# Induction of heme oxygenase-1 by hemin protects lung against orthotopic autologous liver transplantation-induced acute lung injury in rats

**DOI:** 10.1186/s12967-016-0793-0

**Published:** 2016-02-02

**Authors:** Xinjin Chi, Na Guo, Weifeng Yao, Yi Jin, Wanling Gao, Jun Cai, Ziqing Hei

**Affiliations:** Department of Anesthesiology, Third Affiliated Hospital, Sun Yat-sen University, Guangzhou, 510630 Guangdong China; Department of Pathology, Third Affiliated Hospital, Sun Yat-sen University, Guangzhou, 510630 Guangdong China

**Keywords:** Heme oxygenase-1, Acute lung injury, Inflammation, Oxidative stress, Orthotopic autologous liver transplantation

## Abstract

**Background:**

Post-liver transplantation acute lung injury (ALI) severely affects patients’ survival, whereas the mechanism is unclear and effective therapy is lacking. The authors postulated that reperfusion-induced increased oxidative stress plays a critical role in mediating post-liver transplantation ALI and that induction of heme oxgenase-1 (HO-1), an enzyme with anti-oxidative stress properties, can confer effective protection of lung against ALI.

**Methods:**

Male Sprague–Dawley rats underwent autologous orthotopic liver transplantation (OALT) in the absence or presence of treatments with the selective HO-1 inducer (Hemin) or HO-1 inhibitor (ZnPP). Lung tissues were collected at 8 h after OALT, pathological scores and lung water content were evaluated; survival rate of rats was analyzed; protein expression of HO-1 was determined by western blotting, and nuclear translocation of Nuclear factor erythroid 2-related factor 2 (Nrf2) and nuclear factor(NF)-κB p65 were detected by Immunofluorescence staining. The inflammatory cytokines and oxidative indexes of lung tissue were determined.

**Results:**

In lungs harvested at the early stage i.e. 8 h after OALT, Hemin treatment significantly increased superoxide dismutase activities, and reduced malondialdehyde, hydrogen peroxide, interleukin-6, myeloperoxidase, and tumor necrosis factor-α production,which were associated with increased HO-1 protein expression and lower pathological scores and increased survival rate of rats. The underline mechanisms might associate with activation of Nrf2 and inhibition of NF-κB p65 nuclear translocation. However, these changes were aggravated by ZnPP.

**Conclusions:**

Hemin pretreatment, by enhancing HO-1 induction, increased lung antioxidant capacity and reduced inflammatory stress,protected the lung from OALT-induced ALI at early stage of reperfusion.

## Background

Acute lung injury (ALI) is one of the major postoperative complications that are associated with significant morbidity and mortality in patients undergoing orthotopic liver transplantation (OLT) [1, 2]. Lung is the first organ suffered from reperfusion of blood flow which contain lots of inflammatory cytokines such like TNF-α and IL-6 releasing from the ischemia/reperfusion (I/R) liver. This attributes to the high incident rates of pulmonary complication following liver transplantation, but the underling mechanism remains unknown. Several factors have been proposed in the development of OLT-induced ALI, which includes I/R and I/R related inflammation, oxidative stress and endotoxin [3–6]. Among them, oxidative stress and inflammation were suggested as the main cause of ALI at post-OLT [7]. However, the underlying regarding the increased oxidative stress and inflammation in OLT-induced ALI is unclear, and effective means that ameliorate ALI at post-OLT are lacking.

The heme oxygenase (HO) system is the rate-limiting step in the conversion of heme into biliverdin, carbon monoxide (CO), and free iron (Fe^2+^) [8]. Upregulation of HO-1 represents one of the most critical cytoprotective mechanisms that are activated during times of cellular stress, such as inflammation, ischemia, hypoxia, hyperoxia, hyperthermia, or radiation, and it is thought to play a key role in maintaining antioxidant/oxidant homeostasis during multiple cellular injury [9–12]. Overexpression of HO-1 confers protective effects in numerous transplantation models (i.e. heart transplantation, liver transplantation, and kidney transplantation) by enhancing tissue antioxidant capacity, maintaining microcirculation and reducing inflammatory stress [13]. Evidences have shown that induction of HO-1 is cytoprotective in lung disease models both in vivo and vitro, including ALI, ischemia–reperfusion-induced lung injury, lung cancer and asthma [14–16]. However, whether or not induction of HO-1 could protect the lung against OLT-induced ALI has not been study.

We hypothesized that induction of HO-1 may attenuate OALT-induced ALI by the mechanism involving reduction of oxidative stress and inflammation.

## Methods

### Animals

Male Sprague–Dawley rats (weighing 200–250 g) were housed in the Department of Laboratory Animal Center at Sun Yat-Sen University with a specific pathogen-free, laminar flow atmosphere. Animal protocols were approved by the Sun Yat-Sen University Animal Care Committee, and the experiments were performed in adherence to the guidelines provided by the National Institutes of Health for the use of animals in laboratory experiments.

### Orthotopic autologous liver transplantation (OALT) model

Rats were acclimated for one week and food was withheld eight hours prior to operation, but free access to water. Rats were anesthetized with pentobarbital (30 mg/kg body weight) via intraperitoneal injection; under 50 % oxygen delivered using an animal mask. A standard model of OALT was performed as previously described [17]. All the surgical procedures were carried out under sterile conditions.

### Animals groups

The experimental animals were randomly divided into 6 groups (n = 8 per group): sham + saline, sham + Hemin and sham + ZnPP, OALT + saline, OALT + hemin, OALT + ZnPP. Rats, intraperitoneally injected with saline, hemin (30 mg/kg) and ZnPP (20 mg/kg) separately in corresponding groups 24 h before operation, were received celiotomy and vascular separation with or without OALT. Both Hemin and ZnPP may produce other side effects. However, according to the recent study, Hemin has been shown safely and effectively enhance the HO-1 expression [18] and ZnPP also recommended as an inhibitor of HO-1 for relative few off-targets effect [19] and the validity of ZnPP has been checked in our previous study [20]. Because there were no differences in any of the parameters between ZnPP- and hemin-treated rats in the sham groups, the results of groups (sham + Hemin) and (sham + ZnPP) were pooled and were referred to as sham.

### Survival rates

Another 4 groups (n = 18 per group) were observed for survival rates. The survival rates of each group were observed during 36 h from the onset of reperfusion in OALT model.

### Collection of lung tissue

The animals were killed with deeply anesthetized with chloral hydrate (400 mg/kg body weight, i.p.) at 8 h after OALT, the whole lung was removed carefully, and the superior lobe of the right lung was cut and fixed in 10 % formaldehyde and then embedded in paraffin for section. The remaining lung tissue was washed thoroughly with 4 °C normal saline and then stored at −80 °C for further measurements.

### Histological examination

Five-μm thick sections were prepared from paraffin-embedded lung tissue, stained with hematoxylin-eosin (H&E) and assessed for inflammation and tissue damage. And the injury degrees of lung were evaluated by two histologists who were initially blinded to the experiment according to the Derks and Jacobovitz Derks’ standard [21].

### Water content of lung

The wet weights of the middle lobes of the right lung were measured, and the samples were heated at 80∘C for 24 h to reach a constant weight. The water content of the lung was calculated: water content = (lung wet weight − lung dry weight)/lung wet weight × 100 [22].

### Arterial blood gas analysis

The arterial blood partial pressure of oxygen (PaO_2_) of each rat was measured using an iSTAT portable blood gas analyzer (Abbott Laboratories, USA) according to the manufacturer’s instructions [4].

### Lung hydrogen peroxide (H_2_O_2_), malondialdehyde (MDA) concentration and superoxide dismutase (SOD) activity

Lung tissues were made into a homogenate with normal saline, frozen at −20 °C for 5 min and centrifuged for 15 min at 3000*g*. The supernatant was collected for further analysis. The levels of H_2_O_2_ were measured using a kit (Keygen Biotech. Co., Ltd., Nanjing, China).The tissue content of MDA was determined by the TBA method (Jiancheng Bioengineering Ltd, Nanjing, China). The content of MDA in lung tissue was calculated as millimole per milligramme of protein. SOD activity was evaluated with an SOD detection kit according to the manufacturer’s instructions (Jiancheng Bioengineering Ltd, Nanjing, China). The activity of SOD in lung tissue was calculated as U per milligramme of protein.

### Enzyme-linked immunosorbent assay

IL-6 and TNF-α level in lung tissue were measured according to the manufacturer’s instructions of enzyme-linked immunosorbent assay (ELISA) kits which were purchased from the following companies (Jiancheng Bioengineering Ltd, Nanjing, China).

### Detection of myeloperoxidase (MPO) activity

MPO activity is an indicator of polymorphonuclea (PMN) infiltration, which was determined as previously described [23]. MPO activity was defined as the quantity of enzyme that degraded 1 mmol H_2_O_2_ at 37 °C, and it was expressed as U/g wet tissue.

### Immunofluorescence staining

Paraffin sections of lung tissue were washed three times with phosphate-buffered saline (PBS). After fixing with PBS containing 5 % bovine serum albumin and 0.3 % Triton X-100 for 1 h, tissue sections were incubated with anti-Nrf2 (1:100) (Abcam, UK) or anti-NF-κB (1:100) (Abcam, UK) antibodies at 4 °C overnight. After washing with PBS, the slides were incubated with a fluorescently labeled secondary antibody (1:100) (Life technologies, USA) for 1 h at 25 °C. The cover slips were then washed again and mounted using a mounting medium and observed using a fluorescent microscope (Leica, DMLB2, Germany).

### Western blot

The methodology of western blotting analysis has been described previously [7]. Primary antibodies were monoclonal antibody to HO-1 and β-actin (Santa Cruze), the secondary antibody was HP-conjugated IgG antibody (Cell Signaling Technology). Proteins were visualized by an enhanced chemiluminescence assay kit (KGP1125, purchased from Nanjing KeyGen Biotech. Co., Ltd.) and the levels of proteins were normalized with respect to β-actin band density [17].

### Statistical analysis

Data are expressed as mean ± standard deviation. Biochemical assays were performed in triplicate for each specific sample. Therefore, all the data points are means of numbers that themselves are means of triplicate measurements for these parameters. Significance was evaluated using *one*-*way ANOVA* test (SPSS 13.0, SPSS Inc, Chicago, III) followed by *Tukey* post hoc multiple comparisons test for unpaired values. *P* < 0.05 was considered statistically significant.

## Results

### General characteristics

As shown in Table 1, there are no significant differences in body weight, time of the anthepatic phase, and operation time among all groups (*P* > 0.05) (Table 1).

**Table 1:** Basic information of the animals

Group	Body weight (g)	The time of anhepatic phase (min)	Operative times (min)
Sham	225.8 ± 18.5	–	–
Saline + OALT	228.3 ± 14.4	20.2 ± 0.7	57.5 ± 2.6
Hemin + OALT	236.5 ± 15.9	20.2 ± 0.4	58.3 ± 1.5
Znpp + OALT	233.8 ± 20.4	20.3 ± 0.8	57.7 ± 2.2

### Post-OALT lung histologic changes under light microscopy

**Fig. 1:**
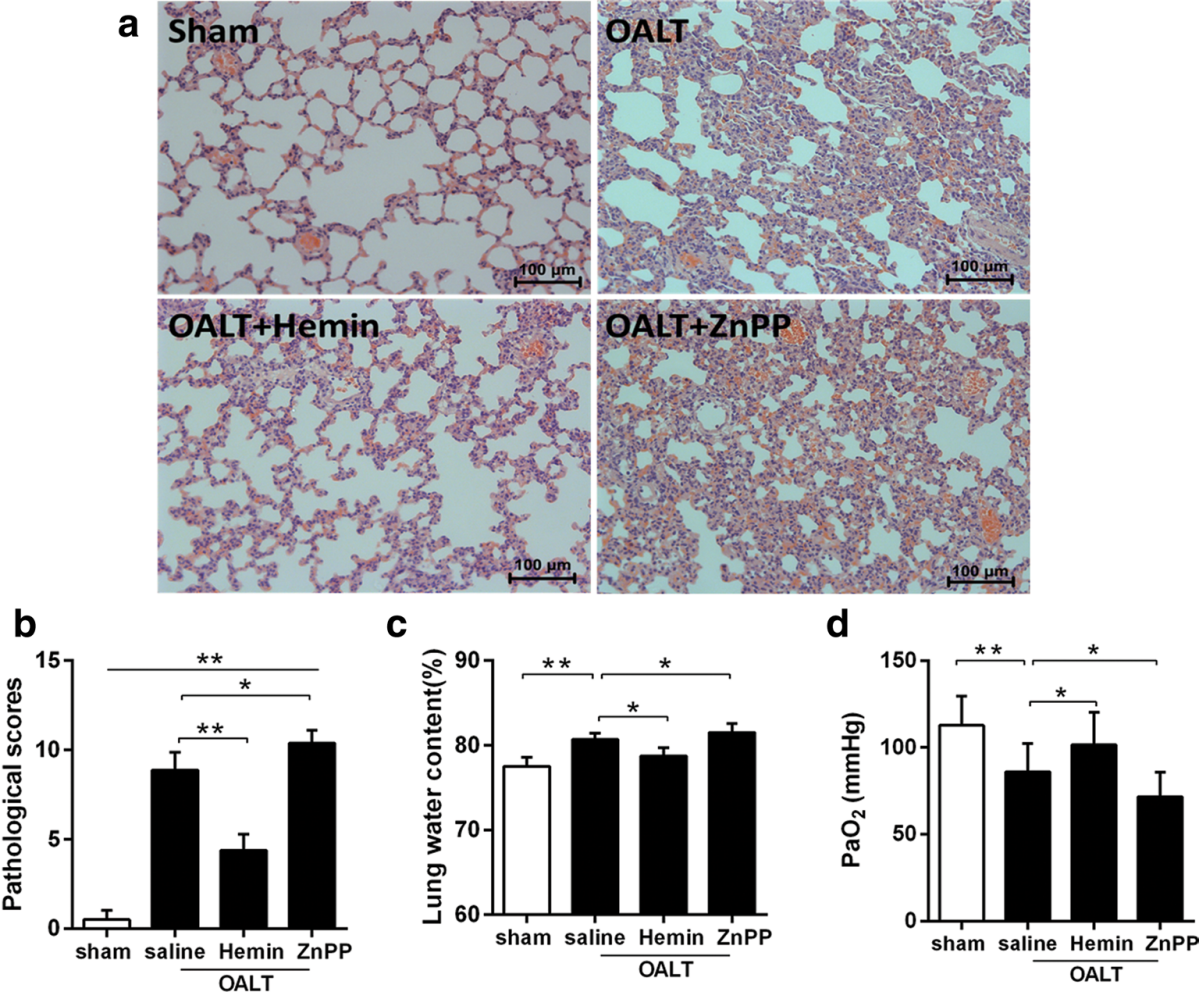
Histopathologic analyses and water content of lung and PaO_2_ level after orthotopic autologous liver transplantation (OALT). HE-stained lung tissue sections from the sham, OALT + saline, OALT + Hemin and OALT + ZnPP groups at 8 h (**a**) after reperfusion (×200). Rats, intraperitoneally injected with saline, hemin (30 mg/kg) and ZnPP (20 mg/kg) separately in corresponding groups 24 h before operation, were received celiotomy and vascular separation with or without OALT. Also, lung injury was assessed by Derks and Jacobovitz Derks’ standard (**b**). Lung water content in lungs (**c**). Level of PaO_2_ (**d**). The results were expressed as the mean ± SD, n = 8 per group. *P < 0.05, **P < 0.01, *one*-*way ANOVA* with *Tukey test*

As shown in Fig. 1. Eight hours after OALT, lung in rats from sham group displayed normal lung tissue morphology with slight inflammatory infiltration with the lowest histopathologic scores among groups (Fig. 1a, b). While lung in rats from model groups displayed severe damage manifested as increased infiltration of polymorphonuclear and mononuclear inflammatory cells into the intra-alveolar and interstitial spaces that were associated with increased interstitial edema and pulmonary architecture damage as well as highest histopathologic scores (*P* < 0.01 vs. *sham*) (Fig. 1b). All these changes were reversed by hemin (*P* < 0.01 vs. saline + OALT group), which were deteriorated by ZnPP (*P* < 0.05 vs. saline + OALT group).

### Water content in lung, PaO_2_ level and the survival rate after OALT

**Fig. 2:**
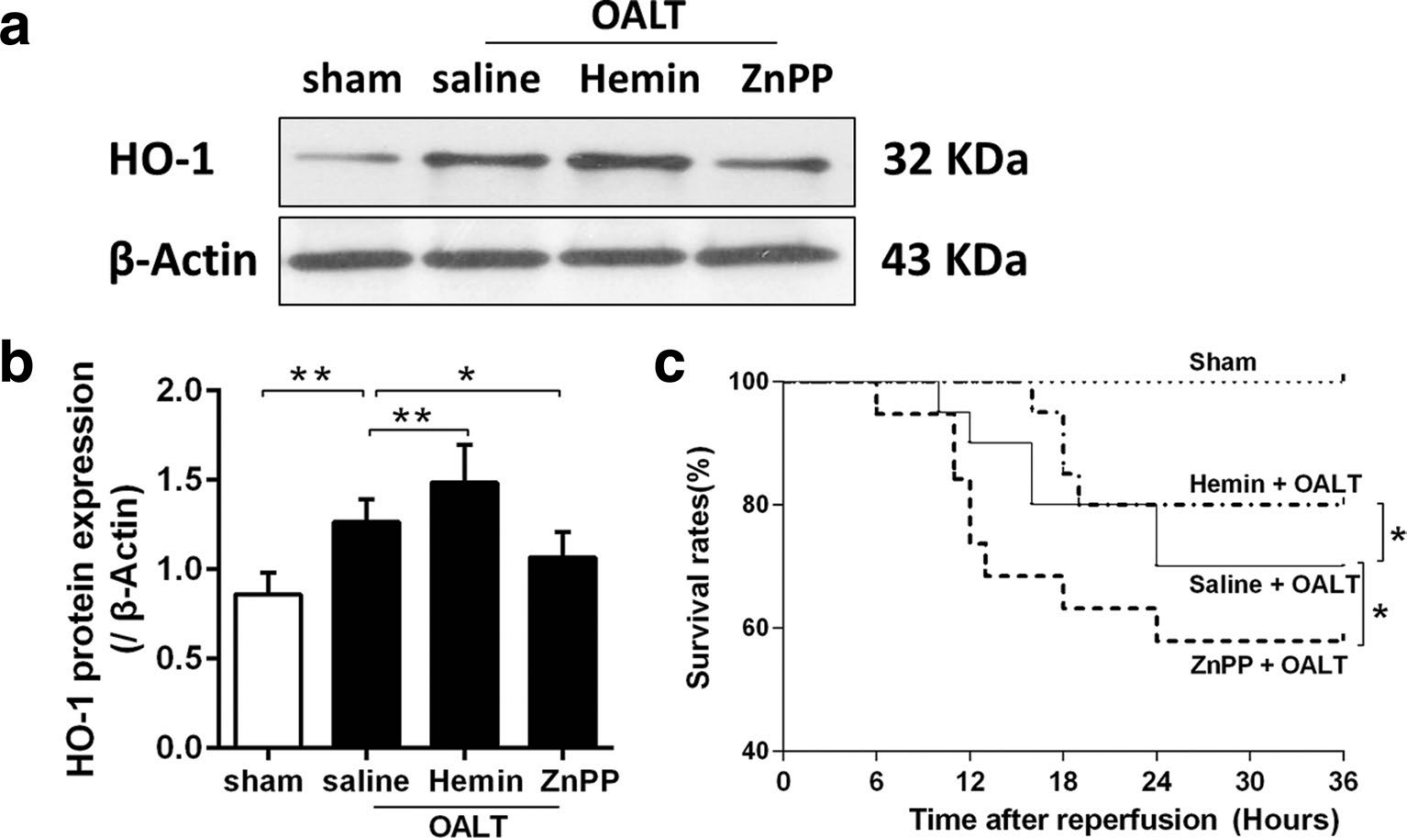
Hemin-increased lung HO-1 expression and survival rates in rats with OLT. Representative western blots analysis (**a**, **b**) of the expression of HO-1 in lung tissue of groups sham, OALT + saline, OALT + Hemin and OALT + ZnPP groups at 8 h after reperfusion. Rats, intraperitoneally injected with saline, hemin (30 mg/kg) and ZnPP (20 mg/kg) separately in corresponding groups 24 h before operation, were received celiotomy and vascular separation with or without OALT. β-actin was run as an internal standard. The results were expressed as the mean ± SD, n = 8 per group. The survival rates during 36 h after reperfusion; n = 18 per group (**c**). *P < 0.05, **P < 0.01, *one*-*way ANOVA* with *Tukey test*

Increased vascular permeability characterizes ALI pulmonary edema and renders fluid balance in the injured lung which is especially sensitive to hydrostatic pressure changes. To determine the degree of pulmonary edema, we detected the wet/dry ratio which reflects the water content of lung. At post-OALT, water content in lung in model group was higher than that in sham group (*P* < 0.01). Hemin pretreatment significantly reduced post-OALT lung water content, while this change was deteriorated by ZnPP (*P* < 0.05 vs. saline + OALT group) (Fig. 1c). PaO_2_ was measured to evaluate pulmonary blood gas barrier dysfunction. PaO_2_ was lower in model group (*P* < 0.01, vs. sham group), and higher in rats pretreated with hemin than that in group sham (Fig. 1d). In order to determine the long term effects of the hemin treatment, we investigated 36 h survival rate for all experimental rats. As shown in Fig. 2c, hemin pretreatment could effectively increase the survival rate of rats after OALT, while ZnPP had the opposite effects.

### HO-1 and Nrf2 protein expression after OALT

**Fig. 3:**
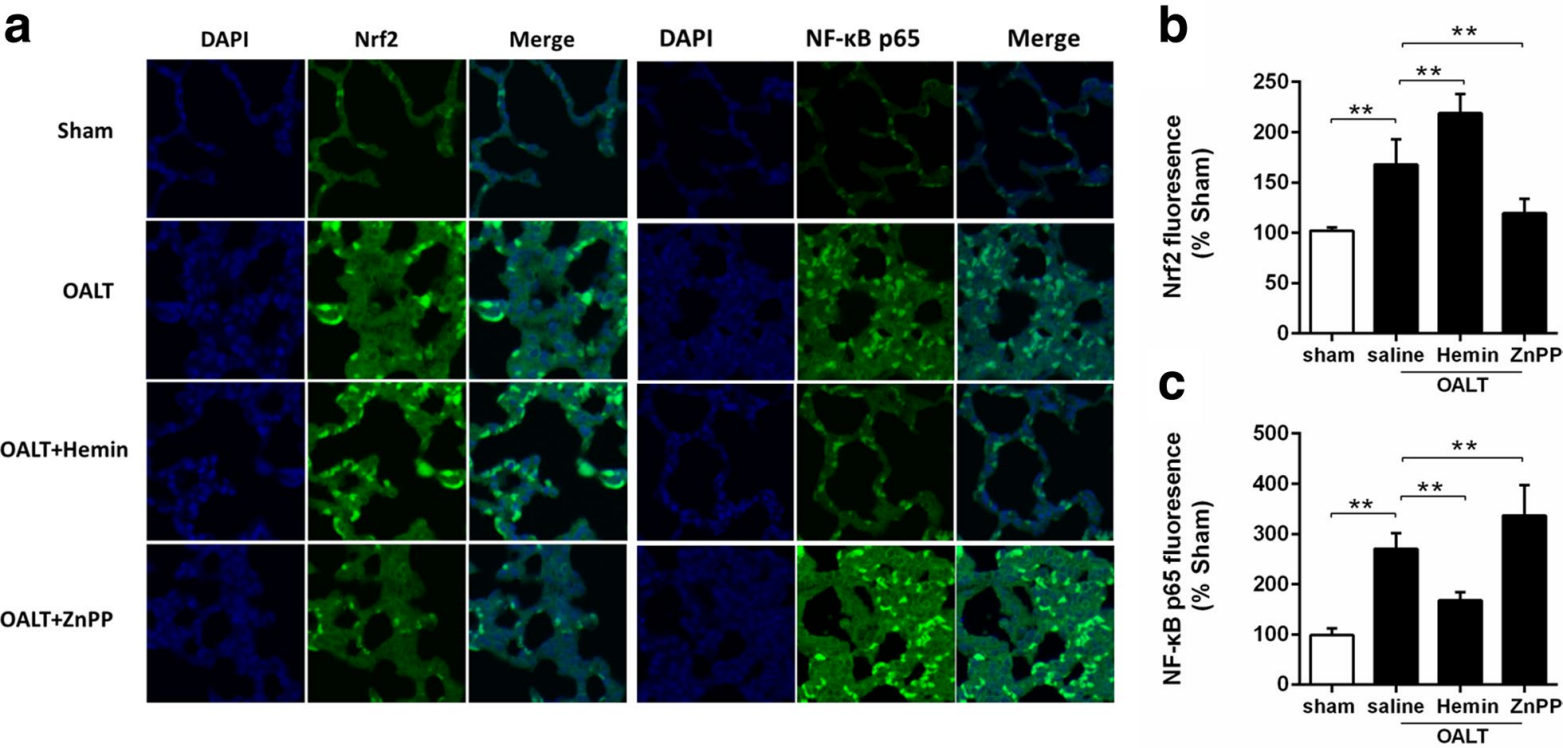
Hemin-induced activation and inactivation of Nrf2 and NF-kB p65 in lung, respectively, in rats with OALT. Immunofluorescent staining of Nrf2 (**a**, **b**) and NF-κB P65 (**a**, **c**) using lung tissue sections of groups sham, OALT + saline, OALT + Hemin and OALT + ZnPP groups at 8 h after reperfusion. Rats, intraperitoneally injected with saline, hemin (30 mg/kg) and ZnPP (20 mg/kg) separately in corresponding groups 24 h before operation, were received celiotomy and vascular separation with or without OALT. The results were expressed as the mean ± SD, n = 8 per group. *P < 0.05, **P < 0.01, *one*-*way ANOVA* with *Tukey test*

As a stress sensing genetic transcription factor, Nrf2 appears to be a master regulator of cellular responses to oxidative damage and other stressful conditions. And the antioxidant response signaling mediated by Nrf2 is called “the primary cellular defense against the cytotoxic effects of oxidative stress”. We assayed the levels of Nrf2 activation and the expression of its downstream antioxidant enzyme HO-1 for evaluating the ability of self-defense which always reflects the resistance to acute injury. At post-OALT, HO-1 (Fig. 2) and Nrf2 (Fig. 3a, b) protein expression in lung tissue were significantly increased in the model group relative to sham group (*P* < 0.01). Pretreatment of hemin significantly increased post-OALT HO-1 and Nrf2 protein expression and were further abolished by ZnPP (*P* < 0.05 vs. saline + OALT group).

### Post-OALT NF-κB P65 protein expression in lung

As an important inflammatory mediator, NF-κB is used to be understood as a protein responsible for cytokine production and cell survival. To determine whether changes of HO-1 could act on this central pro-inflammatory pathway, we measured NF-κB p65 nuclear translocation using immunofluorescence method. At post-OALT, phosphorylation of NF-κB p65 in lung was significantly enhanced in model group, indicating an increased NF-κB nuclear translocation (Fig. 3a, c). We therefore assessed the nuclear translocation of NF-kB p65 subunit after treatment with Hemin and ZnPP. As a result, Hemin pretreatment significantly reduced NF-κB p65 phosphorylation/NF-κB nuclear translocation, while this change was cancelled by ZnPP.

### The alteration of lung TNF-α, IL-6, MPO activities after OALT

**Fig. 4:**
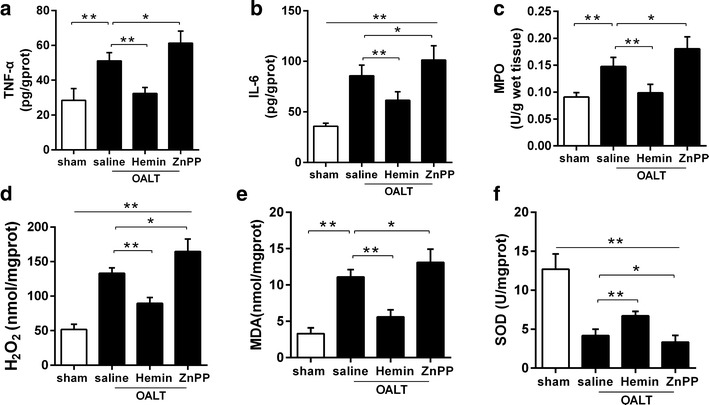
Hemin-induced augment of SOD activity and reduction of MDA, H_2_O_2_, IL-6, MPO and TNF-α production in lung in rats with OALT. The NF-κB downstream inflammation cytokines TNF-α (**a**) and IL-6 (**b**) were detected by ELISA assay. MPO (**c**), H_2_O_2_ (**d**), Malonaldehyde (MDA) (**e**) and the activity of superoxide dismutase (SOD) (**f**) in the lung tissue were detected by assay kits. The results were expressed as the mean ± SD, n = 8 per group. *P < 0.05, **P < 0.01, *one*-*way ANOVA* with *Tukey test*

NF-kB p65 subunit initiated pro-inflammatory cytokines such as TNF-α and IL-6 production, which play a key role in inflammatory response in injured lung. MPO is used as an inflammatory factor of identifying inflammation in the pathological progress of ALI, which in turn may indicate a risk for lung injury. At post-OALT, TNF-α, IL-6 and MPO in the lung were significantly increased in model group compare to sham group (*P* < 0.05). Hemin pretreatment significantly reduced TNF-α, IL-6 and MPO after OALT (*P* < 0.01 vs. saline + OALT group). These changes were abolished by ZnPP (*P* < 0.05 vs. saline + OALT group) (Fig. 4a–c).

### Changes of SOD, MDA and H_2_O_2_ after OALT

Oxidative stress markers SOD, MDA and H_2_O_2_ were examined to reflect oxidative damage degreed of the injured lung. After OALT, in the lung in rats from model group, MDA and H_2_O_2_ were significantly increased while SOD was significantly reduced than those in sham group (*P* < 0.05). Hemin pretreatment significantly reduced MDA and H_2_O_2_, and increased SOD after OALT (*P* < 0.01 vs. saline + OALT group). These changes were attenuated by ZnPP (*P* < 0.05 vs. saline + OALT group) (Fig. 4d–f).

## Discussion

In the current study, we have demonstrated that up-regulation of HO-1 by its activator hemin, through reducing NF-κB p65 activation and enhancing Nrf2, reduced lung oxidative stress (by increasing SOD activities and reducing MDA, H_2_O_2_) and inflammation (by decreasing TNF-α, IL-6, and MPO), and subsequently attenuated OALT-induced ALI and finally increased the survival rate of rats.

Since the publication of the first orthotopic rat liver transplantation (OLT) in 1979 by Kamada et al. [24]., liver transplantation clinical progress has been largely supported by the develop of animal models. And model developed by Kamada has remained the gold standard despite various proposed alternative techniques [25]. However, the broader use of the Kamada method is limited by its steep learning curve [26], and the successful rate of this model is relative low to the novice. On the other hand, during liver transplantation, the lung is injured by ischemia–reperfusion, immunological rejection and infection. In the current study, we used our well established OALT model which was modified from the OALT model developed by Zhao F and his coworker [27], the model is simple and easy-to-establish, more importantly, it simulated the whole process of clinical liver transplantation and was adopted because it avoided the effects of infection and immune suppression on acute lung injury. Such a model allows investigation of relative warm ischemia and reperfusion injury and simulates the main hemodynamic processes during perioperative period of liver transplantation, in which the anhepatic phase can be controlled without the development of the complications of immunological rejection and infection [28, 29] in orthotopic liver transplantation using the cuff technique.

Several factors have been proposed in the development of OALT-induced ALI, which includes ischemia reperfusion (I/R) and the I/R related inflammation, oxidative stress and endotoxin [3–6]. However, data from different research groups were contradictory. Lung injury was significantly increased in OALT model group with significantly enhanced lung oxidative stress and inflammation, indicating that ALI observed in the current study were induced by OALT and that this OALT-induced ALI was due to, at least in part, increased oxidative stress and inflammation.

Oxidative stress has been suggested to play a critical role in the pathologic of ALI [30]. In the current study, in OALT model, lung SOD activities were reduced while MDA and H_2_O_2_ were increased after OALT, indicating an increased oxidative and reduced anti-oxidative state in the lung. These changes were attenuated by hemin treatment that was associated with attenuated ALI after OALT, suggesting that hemin may ameliorate OALT-induced ALI by reducing oxidative stress in the lung. Indeed, hemin is the substrate of HO-1, also known as a physiological inducer of HO-1, can increase both HO-1 mRNA and protein levels. HO-1 is now been well accepted as a potent mediator in the upregulation of antioxidant capacity in various organs including the lung [31, 32], it has been considered as a major inducer of HO-1 expression, heme levels have been reported to contribute to the development of ALI and associated survival of mice [33]. HO-1 catalyzes the first and rate-limiting step in heme degradation into equimolar amounts of CO, iron, and billiverdin, all these downstream targets are critical for survival of mice for their different function in ALI. Biliverdin, a potent endogenous antioxidant [34], with recently recognized anti-inflammatory properties [35], while iron is sequestered by ferritin, leading to additional antioxidant [36] effects. CO has numerous biological functions, including anti-inflammatory properties [37], and shares many similarities with nitric oxide (NO). These enzymatic products of HO-1 are in part responsible for HO-1 action. Interestingly, in the current study, we found that increased of HO-1 by hemin enhanced Nrf2 protein expression, while inhibition of HO-1 by ZnPP reduced Nrf2, suggesting that HO-1 may regulate Nrf2, as it is well accepted that Nrf2 serves as the upstream of HO-1 [38]. Results from our current study provided evidence that HO-1 may regulate Nrf2 in a positive feedback manner. However, the pathway that HO-1 regulates Nrf2 is needed to be further investigated, and to more accurately control the experiment condition, HO-1 sh-RNA and overexpression adenovirus will be involved in our future in vitro study. Futhermore, HO-1 mutant mice have been shown exhibited disrupted lung alveolar structure [39] and HO-1^−/−^ mice exhibited increased susceptibility to oxidative stress [40]. In contrast, Dennery and colleagues recently demonstrated that HO-1^−/−^ mice were surprisingly resistant to hyperoxia and that adenoviral overexpression of HO-1 in HO-1^−/−^ mice worsened lung injury [41]. It’s of interesting to study HO-1 k.o. mice in our model to clarify the controversial relationship between HO-1 and OALT-induced ALI in the future.

Accumulating evidences show that HO-1 confers its cytoprotective effects not only by increasing antioxidative capacity but also by reducing inflammation [42, 43]. In the current study, hemin pretreatment reduced lung NF-κB p65 activation accompanied by reduced inflammation (reduced TNF-α, IL-6, and MPO) in the lung after OALT, Indicating that HO-1 induction attenuated post-OALT ALI by reducing inflammation in the lung. In addition, in our current study, superoxide dismutase (SOD) activity was determined. SOD controls the level of superoxide in the extracellular space by catalyzing the dismutation of superoxide into hydrogen peroxide (H_2_O_2_) and molecular oxygen. Meanwhile, the SOD enzyme reacts with H_2_O_2_ in a peroxidase reaction which is known to disrupt enzymatic activity [44]. HO-1 activation could effectively reduce the production of H_2_O_2_ and then remove restrain to SOD activity, leading to an elevation of SOD activity.

ALI and its more severe form, the acute respiratory distress syndrome (ARDS), are common complications of major liver surgery including liver transplantation, which contributes to perioperative morbidity and mortality. Our previous study showed that 58.2 % of patients (91 patients in total) suffered from pulmonary complications after OLT (orthotopic liver transplantation), and about 27.5 % of them suffered from ALI, and 5.5 % of them endured ARDS [45]. However, we found there was a high incidence rate about pathological change in the rat OALT model, which can’t be detected in clinical, indicating that pathological change might be happen earlier than pulmonary functional change. Whether the pathological change of lung plays a more sensitivity role in clinical ALI diagnosis will be attractive in our further study by developing the method to detect the pathological change.

Of note, as most bacteria need iron as a vital nutrient, the ability of bacterial pathogens to acquire iron during infection is essential for many organisms to cause disease [46]. A very limited amount of iron is available to an invading pathogen within the host. Since much of the intracellular iron is associated with hemin [47], whether administration of hemin could increase the chance of bacterial infection in the liver transplantation-induced ALI which has been recognized as a bacteria-free inflammation progress is very interesting issue and need further investigation.

## Conclusions

In summary, to our knowledge, our study demonstrated for the first time, that induction of HO-1 through increasing Nrf2 and deactivating NF-κB reduced oxidative stress and inflammation in the lung after OALT, which subsequently attenuated OALT-induced ALI at the early stage of reperfusion. HO-1 might therefore represent as novel therapeutic targets in the treatment of OALT-induced ALI. Furthermore, elucidating the underlying mechanism, especially the role of HO-1, in attenuating OALT-induced ALI may provide important information for the development and the optimization the use of clinical drugs in the treatment of liver transplantation-induced ALI.
